# Phenylketonuria Patients’ and Their Caregivers’ Perception of the Pandemic Lockdown: The Results of a National Online Survey

**DOI:** 10.3390/children9020131

**Published:** 2022-01-19

**Authors:** Dariusz Walkowiak, Bożena Mikołuć, Renata Mozrzymas, Łukasz Kałużny, Bożena Didycz, Dorota Korycińska-Chaaban, Michał Patalan, Joanna Jagłowska, Agnieszka Chrobot, Rafał Staszewski, Jarosław Walkowiak

**Affiliations:** 1 Department of Organization and Management in Health Care, Poznan University of Medical Sciences, 60-356 Poznan, Poland; 2 Department of Pediatrics, Rheumatology, Immunology and Metabolic Bone Diseases, Medical University of Bialystok, Waszyngtona Str. 17, 15-274 Bialystok, Poland; bozenam@mp.pl; 3 Research and Development Center, Regional Specialist Hospital, Kamieńskiego Str. 73a, 51-124 Wroclaw, Poland; renata.mozrzymas@gmail.com; 4 Department of Pediatric Gastroenterology and Metabolic Diseases, Poznan University of Medical Sciences, Szpitalna 27/33, 60-572 Poznan, Poland; lukasz@jerozolima.poznan.pl (Ł.K.); jarwalk@ump.edu.pl (J.W.); 5 Outpatient Metabolic Clinic, University Children’s Hospital, 30-663 Cracow, Poland; bozenadidycz@wp.pl; 6 PKU Polyclinic, Institute of Mother and Child, Kasprzaka 17A, 01-211 Warsaw, Poland; chaaban@wp.pl; 7 Department of Pediatrics, Endocrinology, Diabetology, Metabolic Diseases and Cardiology, Pomeranian Medical University, Unii Lubelskiej 1, 71-252 Szczecin, Poland; mpatalan@hotmail.com; 8 Department of Pediatrics, Hematology and Oncology, Medical University of Gdansk, Dębinki Str. 7, 80-211 Gdansk, Poland; chabros_joanna@hotmail.com; 9 Voivodship Children Hospital, Chodkiewicza Str. 44, 85-667 Bydgoszcz, Poland; aga.chrobot@wp.pl; 10 Department of Hypertension, Angiology and Internal Medicine, Poznan University of Medical Sciences, Długa Str. 1/2, 60-356 Poznan, Poland; rstaszew@ump.edu.pl

**Keywords:** phenylketonuria, PKU, remote medicine, COVID-19 pandemic

## Abstract

The first pandemic lockdown dramatically impacted many aspects of everyday life, including healthcare systems. The purpose of this study was to identify problems of patients with phenylketonuria (PKU) and their parents/caregivers during that time. We aimed to analyse potential differences in the self-reported compliance and characteristics of contacts with a doctor/dietitian before and during the pandemic lockdown and the perception of access to special food and opinions on remote contacts between a particular group of respondents. All participants (*n* = 614) were asked to complete an online questionnaire that consisted of 31 questions on pandemic-related events and circumstances which may have directly or indirectly impacted health and treatment. The people who completed the survey were divided into three groups: parents of PKU children (*n* = 403), parents of PKU adults (*n* = 58) and PKU patients older than 16 years (*n* = 153). The differences among the three analysed groups were found in the number of contacts, the way of contacting a doctor/dietitian during the pandemic and satisfaction with remote contact. Caregivers of children with PKU reported better therapy compliance, more frequent contacts with specialists and more satisfaction with remote visits than adult patients. We also observed a relationship between satisfaction from remote contact and self-reported frequency of contacts with a doctor/dietitian, as well as a relationship between satisfaction from remote contact and recommended blood Phe levels reported by both patients and caregivers. Travel time exceeding three hours from the respondents’ location to their doctor was associated with higher odds of their recognition of remote contact as a method of PKU treatment only in the group of caregivers. In the caregiver groups, the reported worse access to low-Phe products during the lockdown was linked to the perceived difficulty of maintaining the diet. However, such a relationship was not found among patients. In conclusion, significant differences in the perception of the pandemic lockdown and its impact on health and treatment-related issues were found.

## 1. Introduction

According to the 2020 NORD survey [1] of those affected with rare disorders, their caregivers and families, 79% had a medical appointment cancelled due to COVID-19, 83% were offered a telephone or video call as an alternative to an in-office appointment and as many as 62% were concerned with medication supply shortages. The results of the Rare Diseases Ireland survey [2] indicated that 73% of the respondents were worried about their health, 53% of scheduled appointments across all healthcare settings had been cancelled and 26% of patients encountered difficulties accessing medicines and medical supplies. Furthermore, the Italian National Institute of Health Rare Diseases COVID-19 Working Group [3] reported that 52% of the respondents had given up hospital treatment to prevent exposure to infection, while 46% had problems with their continuity of care or treatment due to outpatient facilities being closed by government decree.

The confirmation of the first Polish case of SARS-CoV-2 infection came on 4 March 2020. The state’s government imposed various types of lockdown control measures; on 12 March, primary, secondary and tertiary schools all over the country ceased to function and subsequently went online. On 15 March, the borders of Poland were closed for foreigners, and on 25 March, the freedom of movement for Polish citizens within Poland was restricted chiefly to basic grocery shopping, visiting a doctor or a pharmacy or going to work that could only be performed in person. On 1 April, minors were forbidden to move unattended in public spaces, and restrictions were introduced in the operation of shops and services. Meanwhile, the activities of institutions and companies were limited, with employees delegated to remote work. Dismissals of employees have also started in many companies. The situation in health care units was extremely complicated. At the end of January, the testing of patients for the presence of coronavirus began, and in February, the first Polish patients suspected of SARS-CoV-2 infection were hospitalised. In many localities, access restrictions have been introduced, first for visitors, and later also for patients. Patients themselves also began to limit their contacts with health care units. Some of the hospitals that used to care for phenylketonuria (PKU) patients so far have been included in the network of hospitals intended for patients with coronavirus. The difficulties with the personal access of PKU patients to their specialist doctors and nutritionists began in February and March, depending on the region of the country and the specifics of a particular hospital.

Indeed, COVID-19 has impacted the treatment of a variety of conditions. This was felt primarily by patients and their families but also by healthcare professionals as well as the healthcare system as a whole. The situation impacted health services in terms of the diagnosis and ensuing therapy, with healthcare professionals faced with hitherto unknown challenges that included switching from in-person to telemedicine meetings with phone- and/or video-aided visits, the introduction of social distancing and other safety measures to minimise the risk of COVID-19 transmission and coping with the shortages of personal protective equipment [4,5,6,7]. Moreover, vacancies among many medical professions became more evident due to increased needs in the healthcare system.

In a recently published national online survey on how PKU patients in Poland perceive health and therapy-related issues [8], we documented that 9.5% of our respondents failed to perform blood phenylalanine (Phe) tests throughout the examined six-week pandemic period (16 March–30 April 2020), with 21.3% reporting an increase in their blood Phe level as opposed to 15.3% who reported a decrease. Moreover, 26.1% of subjects found contacting their doctor or dietitian more difficult, while 39.3% perceived their access to the products necessary in their diet as restricted, but 63.4% were happy with the distance contact with their PKU specialist. Following the requirements of the diet more faithfully was associated with more satisfaction with remote contact, fewer problems subjectively experienced in contact with the doctor and lower odds of failing to undergo a Phe test. Self-assessed high stress was linked to higher odds of subjectively perceived limited access to Phe-free formulas and low-Phe products, as well as higher Phe concentrations and complaints other than those connected with PKU. These patients were also characterised by inadequate dietary compliance and had more difficulty contacting a PKU-treating centre [8].

The present study aimed to analyse potential differences in the self-reported compliance and characteristics of contacts with their doctor/dietician before and during the pandemic lockdown and the perception of remote access to special food and opinions of parents of PKU children and adults, as well as PKU patients older than 16 years who responded to the above-mentioned survey [8] or its continuation. It was hypothesised that different respondents would experience the pandemic lockdown and the consequences of the restrictions in different ways.

## 2. Materials and Methods

The distribution of invitations for PKU patients and their caregivers to take part in an anonymous online survey was carried out by phone, electronic post or by sms, as well as during planned visits to PKU specialists in ten specialised PKU-treating centres all over Poland. We also requested those responsible for running local PKU communities’ websites to place the information about our survey there. Moreover, the information was shared by the patients themselves on Facebook and by telephone. This was confirmed in conversations with the patients, who informed their doctors about having completed the questionnaire. We collected our data in the time period 15 June–20 July 2020, except for one PKU treating centre that communicated with their patients via website only, which led to delays in survey distribution, so we decided to include questionnaires completed until the end of August. The inclusion criteria were a diagnosis of PKU reached by newborn screening, dietary recommendations adhered to since the diagnosis was made and systematic care in a PKU-treating centre. The exclusion criterion was persons aged under 16 years or unknown.

The respondents were divided into two subsets, patients and their caregivers, who were further divided into those caring for patients under 18 years of age and those caring for adults. The latter group consisted of only 58 caregivers; therefore, in many analyses, they were combined with the group of caregivers of underage patients. Needless to say, we made sure that this was logical, justified on therapeutic grounds and did not interfere with the results obtained, since the parents’ perspectives may be different from those of adult patients themselves. Moreover, parents’ answers on their adult children’s behalf may point to possible social differences, which we decided not to pursue further as it was not the focus of our study.

The non-validated questionnaire consisted of 31 questions, 27 of which were single choice, 2 multiple choice and 2 open-ended questions [8]. All questions referred to circumstances and pandemic-related events that may have directly or indirectly impacted our subjects’ health and therapy-related issues, as well as their perception of the consequences of the pandemic. We prepared our survey in simple wording, and in most cases provided options to choose from on a 5-point Likert scale, ranging from 1 (strongly disagree/very dissatisfied) to 5 (strongly agree/very satisfied). The following demographics were also collected: the age of the patient, gender, the distance between home and PKU-treating centre, the time needed to travel to the centre and the way of getting there, and in the case of the questionnaire completed on behalf of the patient, we collected information on the respondent (parents or other caregivers). A past history of a COVID-19 infection or quarantine was also of interest to us, as well as how and how often our subjects remotely talked with their PKU specialists or dietitians during or before the pandemic. We also inquired if our subjects encountered any problems in getting in touch with specialists and the frequency of these contacts were analysed. We also inquired about the patient’s blood Phe levels before and during the lockdown, as well as about the problems they encountered maintaining their diet in connection with possible issues with the supply of low-Phe products and Phe-free formulas. Several questions were related to the evaluation of the distance contact with a doctor or a dietitian, the respondent’s interest in the continuation of this type of contact after the pandemic and the option of remote treatment of PKU and other illnesses. We also wanted to know if our subjects used to leave home during this period and if so, why, as well as about their contacts with other PKU patients. In 2 open-ended questions, we encouraged our patients/caregivers to share a detailed opinion of remote visits and the influence of the lockdown (not being able to go out, more duties) on following the diet. The survey was accompanied by information about the aim and methods of our research and the name and the e-mail address of the coordinator of the study.

The data gathered in the questionnaires were checked for quality, completeness and consistency and exported into the statistical package JASP Team (2021), JASP (Version 0.14.1) [Computer software] and STATISTICA v. 13.3 (TIBCO Software, Palo Alto, CA, USA). The results are presented as descriptive statistics, means and standard deviations (SDs), ranges and percentages. The odds ratio (OR) was calculated to compare one group of patients according to different characteristics based on specific feelings, behaviour or opinions, to other respondents. To estimate the precision of the OR, the 95% confidence interval (95% CI) was calculated. Kruskal–Wallis ANOVA and post hoc tests were used to determine statistically significant differences between the three groups. We set up the level of significance at *p* < 0.05.

## 3. Results

We received 620 completed questionnaires, of which 614 were qualified for further analysis, while six were disqualified due to respondents’ age being under 16. Patients aged 16 to 44 years completed 153 questionnaires, while 58 questionnaires were filled in by caregivers of adult patients with PKU and 403 were completed by parents of children (>18 years old). There was a difference in the gender in the group in which patients completed the questionnaires independently and the group in which the questionnaires were filled in by parents of underage patients. Self-reported Phe levels before the pandemic in PKU patients aged under 18, as reported by their parents (group 3), differed from those declared by the other two groups (group 1 and group 2). Respondents from group 3 reported the best results of self-reported blood Phe levels before the pandemic, with 74.4% of them reporting Phe levels as recommended. However, during the pandemic, statistically significant differences in changes of self-reported blood Phe levels were observed only between patients from groups 1 and 3 (Table 1). Respondents from group 3 reported the best results of self-reported blood Phe levels before the pandemic, with 74.4% of them reporting Phe levels as recommended.

Most respondents declared to have contacted their doctor/dietitian by phone before the pandemic, with parents of PKU patients under 18 using telehealth more often than the other groups (Table 2). This was also confirmed by the declared rates of visit frequency during the lockdown, and in this case, these parents contacted the specialists more often. During the lockdown, the most frequently used communication channels were telephone calls, text messages (SMS) and e-mails, with the caregivers of patients under 18 years of age contacting their doctors by phone more often than patients older than 16 years. The other forms of contact were not popular. Group 3 most often reported contacting a PKU specialist by phone before the pandemic, and a statistically significant difference was noticed when group 3 was compared with group 1. A similar difference was found when we asked about remotely contacting a PKU doctor or dietitian during the pandemic lockdown.

During the lockdown, a significant proportion of patients and caregivers reported deterioration in the supply of Phe-free formulas and low-Phe products, but there were no differences between the groups (Table 3). Most of the respondents from all the three groups reported no changes in access to low-phenylalanine products during the pandemic. In group 1 such an opinion was held by 56.9% of respondents, while this opinion was reported by 69.0% and 61.8% of group 2 and 3, respectively. Access to phenylalanine-free formulas was reported as similar to that before the pandemic by 72.5% of respondents in group 1, by 82.8% in group 2 and by 79.2% in group 3. Answering the question concerning the difficulty of sticking to the diet during the pandemic lockdown, respondents in all three groups most often considered it to be the same as before pandemic.

The parents of children under 18 differed significantly from patients in their opinions about remote contact with a specialist, with no difference in the interest in future remote contacts or in the opinions regarding replacing direct contact with a specialist with remote contact for PKU and other diseases (Table 4). Regarding satisfaction with the remote contact in groups 1 and 3, there was a statistically significant difference between them. We did not find differences between groups in the interest in future remote contacts or in admitting the possibility of replacing direct contact with a specialist by remote visits. There was likewise no difference between groups when we asked our respondents if they could imagine a remote visit in the case of other diseases.

We divided our patients into two groups according to their responses to the question about pre-pandemic self-reported Phe levels. The first group included patients who declared their pre-pandemic Phe levels to be as recommended, while the second group’s self-reported Phe levels were above the target range. The OR of being satisfied with remote contact with a PKU specialist for an individual reporting Phe levels before the pandemic within the target range was higher than for those with Phe levels above the target range. Similarly, the caregivers were divided into two groups in line with their responses to the question “Did you use to contact your PKU specialist/dietitian by phone prior to the pandemic?” (see Table 2). The first one comprised those who claimed to have had frequent phone contact with their doctor (answer: regularly), while the remainder constituted the second group. The OR of being satisfied with remote contact with a PKU specialist for a caregiver having regular contact in the past was higher than for one with no or only infrequent contact (Table 5).

Travel time exceeding three hours from the respondents’ location to their PKU-centre was linked to a higher OR of their acceptance of remote contact as a method of PKU therapy only in the group of caregivers (Table 6).

In the caregiver groups, the reported worse access to low-Phe products during the lockdown was linked to the perceived difficulty of maintaining the diet (Table 7). However, such a relationship was not found among patients.

## 4. Discussion

The most important findings of the present study comprise significant differences in self-reported blood Phe levels and satisfaction with remote medical visit between caregivers of children with PKU and adult PKU patients. According to our research, telephone contact was the most common form of communication between PKU patients and caregivers of patients with a doctor and dietitian during the lockdown. We also observed that both patients and caregivers reporting regular contact with their doctor/dietitian and recommended blood Phe levels before the lockdown expressed higher satisfaction with the contact with doctors/dietitians. In addition, we documented the relationship between travel time and the assessment of a remote visit, as well as that between the perception of compliance and self-reported Phe levels in caregivers but not in adult patients.

Patients with PKU as well as other RDs faced various adversities before the pandemic. In the case of PKU, the changes seem significant, as patients had to stay at home, their physical activity changed, and in the case of adult patients and parents of children with PKU, the way of working also changed. Most previous habits became difficult to implement, and many patients were simply forced to change them overnight. Our study found that the self-reported Phe results of only about half of the patients remained unchanged during the lockdown, with the rest reporting either improvement or deterioration in levels. These changes affected all the studied groups of patients. In a recent study [9], we documented decreased frequency of blood tests in paediatric PKU patients in the pandemic lockdown. Phe concentrations obtained by patients during the lockdown did not become higher; rather the subjects previously identified as non-compliant more often failed to do tests during the assessed period, while those subjects who earlier showed compliance more often performed one test only. However, due to several circumstances, we could not directly compare these results. First, the Phe levels in adults were not analysed, and all paediatric subjects with PKU under the care of six centres, not only those who completed online questionnaires, were included in the study. Our findings suggest that the results of those patients who completed the questionnaire themselves were not satisfactory, even before the pandemic, and were statistically significantly different from the results of the patients under the care of their parents.

A study by Rovelli et al. [10] proved that in patients aged 4–12, it was possible to reduce the level of non-compliance during the first lockdown period in Italy, with significant reductions in the mean blood Phe concentrations of patients over 12. Zubarioglu et al. [11] observed a statistically significant drop in the number of tests performed, with a concomitant improvement in the results of those who conducted the tests during the pandemic lockdown in 2020. Oge Enver et al. [12] found that 74% of Turkish patients with inherited metabolic diseases (IMD) on a diet during the lockdown “did not have a problem about reaching their special formulas and low-protein dietary products”, whereas our study revealed that 37.9% of patients experienced a deterioration in the supply of low-Phe products. It seems that one of the basic problems, apart from the anxiety related to the general situation and concern for one’s health, was the issue of the supply of food products necessary to follow the diet. Many patients experienced various limitations in this respect, with shortages of low-Phe products in particular, which is a significant problem. Interestingly, patients/caregivers reported more problems with low-Phe products than with Phe-free formulas, but it was less difficult for most patients to adhere to the diet during the lockdown than before. Assuming that this was the reality, that is, due to objective or subjective causes, e.g., pandemic-related stress and not exclusively the patients’ feelings, it could have impacted their diet and deteriorated problems related to vitamin and mineral intake already observed in the pre-pandemic period [13,14,15,16,17]. Moreover, these issues together with general changes in food consumption observed during the pandemic could have influenced energy and metabolic balance, increasing overweight and obesity problems observed in patients with PKU [18,19]. Unfortunately, it cannot be concluded that patients were unaffected by experiencing a shortage of the products necessary to prepare the diet, even if this was due to the greater amount of time spent at home; many believed that maintaining the diet was not a problem. Our research shows that there is a relationship between self-reported poorer access to low-Phe products and self-reported compliance.

Earlier studies have shown an increase in stress related to the spread of the coronavirus in the families of patients, impacting the habits of patients regarding walking outdoors [20]. A significant proportion of patients changed their behaviour to adapt to the epidemic threat. An additional circumstance was also the restriction of patients’ access to various forms of therapy and rehabilitation. Lampe et al. hypothesised that the continuous disruption of the appropriate follow-up of patients with IMD might lead to compromising their clinical condition and worsen the course of the metabolic disease from not only a medical but also a psychological and social point of view [21]. However, patients with PKU are less subjected to such influences than most patients with IMD. Tummolo et al. [22] reported a moderate influence of the COVID-19 pandemic on a relatively numerous cohort of IMD patients, which included children and young adults, but Chung et al. established that patients with rare diseases had a 3.4 times higher OR of hospital death from COVID-19-related causes than the general population [23].

This study has some limitations. More caregivers of children than adult subjects with PKU and caregivers of adult patients participated in the survey, and their opinions refer to the relatively short six-week lockdown. However, from a longer perspective, the first wave could have exerted the most significant effect due to uncertainty. Due to the anonymous character of the questionnaire, we cannot refer to any objective measures of epidemiological characteristics, nutrition and compliance, but it may have resulted in more sincere responses. The survey nature of the study makes it impossible to verify the truthfulness of the answer, including self-reported blood Phe levels, before the pandemic and changes during the pandemic. The strength of the present study is the large sample size, with a very high response rate. In our study, most patients aged 16 and over were women. The observed differences in the gender structure of the studied groups most likely resulted from the emphasis placed on the prevention of maternal PKU, which may explain the greater participation of adult women and balanced gender structure in the other groups. As both patients and their caregivers were invited to complete the questionnaire, the obtained results concern a broad population of families of people suffering from PKU. Despite the high rate of return of the questionnaires, the numbers of individual groups completing the questionnaire are different. Moreover, the situation of adults on behalf of whom the parents have filled in the questionnaire is not entirely clear. However, we decided to include the group of their parents because this group of respondents (and their perspectives) seemed important. Additionally, it might differ from that of caregivers of young patients and of adult patients.

## 5. Conclusions

Caregivers of children with PKU reported better therapy compliance, more frequent contacts with specialists during the analysed six-week pandemic period and more satisfaction with remote visits than adult patients. There was also a relationship between satisfaction with remote contact and self-reported frequency of contacts with a doctor/dietitian, as well as between satisfaction with remote contact and recommended blood Phe levels in both patients and caregivers. Moreover, the travel time and the feeling of poorer access to low-phenylalanine products were related to the different perceptions of the value of remote contacts and compliance in adult patients and caregivers. Our study also indirectly allows certain insights into the best ways of dealing with potential future emergency periods: more effort should be dedicated to the popularisation among adult patients of the remote mode of contact with specialists. Further studies are necessary to identify the most vulnerable persons and develop an appropriate strategy of medical support for individual patients and caregivers.

## Figures and Tables

**Table 1:** Patients’ characteristics.

Characteristics	1. Patients *n* (%N)	2. Caregivers of Patients over 18 Years Old *n* (%N)	3. Caregivers of Patients under 18 Years Old *n* (%N)	*p* * (Post Hoc Tests for Groups) **
Number of Patients	N = 153	N = 58	N = 403	
Patient gender				**<0.001** (1 vs. 3, *p* < **0.001**)
Female	105 (68.6)	30 (51.7)	183 (45.4)	
Male	48 (31.4)	28 (48.3)	220 (54.6)	
Patients’ age (years of age)				**<0.001** (1 vs. 3, *p***< 0.001**)
Mean age (SD)	28.5 (6.7)	31 (9.6)	6.6 (4.4)	
Median age (IQR)	29 (24–34)	31 (22.3–37)	6 (3–10)	
Age range	16–44	18–53	0.2–17	
Pre-pandemic self-reported blood Phe levels				**<0.001** (1 vs. 3, *p* < **0.001**; 2 vs. 3, *p* = **0.001**)
As recommended	66 (43.1)	29 (50.0)	300 (74.4)	
Slightly too high	71 (46.4)	24 (41.4)	89 (22.1)	
Far too high	16 (10.5)	5 (8.6)	14 (3.5)	
Self-reported blood Phe levels changes during the pandemic lockdown				**0.002** (1 vs. 3, *p* = **0.005**)
Increased considerably	7 (4.6)	2 (3.4)	9 (2.2)	
Increased slightly	26 (17.0)	8 (13.8)	50 (12.4)	
Remained the same	68 (44.4)	27 (46.6)	235 (58.3)	
Decreased slightly	22 (14.4)	8 (13.8)	71 (17.7)	
Decreased considerably	6 (3.9)	2 (3.4)	19 (4.7)	
No tests in lockdown period	24 (15.7)	11 (19.0)	19 (4.7)	

Statistically significant results are marked with boldface. Standard deviation (SD), Interquartile Range (IQR) * Kruskal–Wallis ANOVA. ** Post hoc test shown exclusively for significant differences.

**Table 2:** Details of contact with a PKU doctor/dietitian.

Characteristics	1. Patients *n* (%N)	2. Caregivers of Patients over 18 Years Old *n* (%N)	3. Caregivers of Patients under 18 Years Old *n* (%N)	*p* * (Post Hoc Tests for Groups) **
**Number of Patients**	N = 153	N = 58	N = 403	
**Did you use to get in touch with your PKU dietitian/specialist by phone before the pandemic?**				**<0.001** (1 vs. 3, *p* < **0.001**)
Yes, regularly	27 (17.7)	19 (32.8)	100 (24.8)	
Yes, several times	27 (17.7)	10 (17.3)	141 (35.0)	
Maybe once	19 (12.4)	5 (8.6)	37 (9.2)	
I do not remember	12 (7.8)	2 (3.4)	13 (3.2)	
I did not feel such a need	60 (39.2)	22 (37.9)	109 (27.1)	
There was no such possibility	8 (5.2)	0 (0)	3 (0.7)	
**Before the pandemic, did you use to get in touch with your PKU dietitian/specialist via Skype, video conference or through social media?**				0.31
Yes, regularly	6 (3.9)	4 (6.9)	18 (4.5)	
Yes, several times	7 (4.6)	1 (1.7)	22 (5.4)	
Maybe once	4 (2.6)	3 (5.2)	6 (1.5)	
I do not remember	11 (7.2)	4 (6.9)	13 (3.2)	
I did not feel such a need	85 (55.6)	37 (63.8)	240 (59.6)	
There was no such possibility	40 (26.1)	9 (15.5)	104 (25.8)	
How did you contact your doctor or dietitian during the pandemic lockdown? ***				**0.02** (1 vs. 3, *p* = **0.02**)
Phone	66 (43.1)	33 (56.9)	256 (63.5)	
Videochat	0 (0)	0 (0)	2 (0.5)	
Messenger and WhatsApp	7 (4.6)	1 (1.7)	8 (2.0)	
Facebook	4 (2.6)	0 (0)	1 (0.2)	
Text message	16 (10.4)	7 (12.1)	54 (13.4)	
E-mail	26 (17.0)	6 (10.3)	85 (21.1)	
**Have pandemic restrictions changed how difficult it is to communicate with your specialist doctor/dietitian?**				0.28
Increased considerably	9 (5.9)	3 (5.2)	11 (2.7)	
Increased slightly	39 (25.5)	12 (20.7)	77 (19.1)	
Remained similar	47 (30.7)	13 (22.4)	126 (31.3)	
Decreased slightly	21 (13.7)	5 (8.6)	73 (18.1)	
Decreased considerably	37 (24.2)	25 (43.1)	116 (28.8)	
**Number of contacts with a doctor**				**0.01** (1 vs. 3, *p* = **0.01**)
Mean (SD)	1.2 (1.7)	1.4 (1.7)	1.9 (2.3)	
Median (IQR)	1 (0–2)	1 (0–2)	1 (0–3)	
Range	0–10	0–9	0–12	
Missing data—*n* (%)	5 (3.3)	0 (0)	0 (0)	

Statistically significant results are marked with boldface. * Kruskal–Wallis ANOVA. ** Post hoc test shown exclusively for significant differences. *** Respondents could choose no answer or any number of options. The results do not add up to 100%.

**Table 3:** The views of respondents on availability of special food and their opinion on being on the diet during the pandemic lockdown.

Characteristics	1. Patients *n* (%N)	2. Caregivers of Patients over 18 Years Old *n* (%N)	3. Caregivers of Patients under 18 Years Old *n* (%N)	*p* *
**Number of Patients**	N = 153	N = 58	N = 403	
**How was your access to low-phenylalanine products during the pandemic lockdown?**				0.83
Definitely worse	7 (4.6)	3 (5.2)	26 (6.4)	
Worse	55 (35.9)	14 (24.1)	128 (31.8)	
The same as before the pandemic	87 (56.9)	40 (69.0)	249 (61.8)	
Better	2 (1.3)	0 (0)	0 (0)	
Definitely better	2 (1.3)	1 (1.7)	0 (0)	
**How was your access to phenylalanine-free formulas during the pandemic lockdown?**				0.90
Definitely worse	9 (5.9)	1 (1.7)	10 (2.5)	
Worse	26 (17.0)	9 (15.5)	73 (18.1)	
The same as before the pandemic	111 (72.5)	48 (82.8)	319 (79.2)	
Better	4 (2.6)	0 (0)	0 (0)	
Definitely better	3 (2.0)	0 (0)	1 (0.2)	
**Was it difficult to stick to the diet during the pandemic lockdown?**				0.34
Definitely more difficult	3 (2.0)	0 (0)	6 (1.5)	
More difficult	31 (20.3)	4 (6.9)	33 (8.2)	
The same as before the pandemic	60 (39.2)	28 (48.2)	187 (46.4)	
Easier	32 (20.9)	15 (25.9)	102 (25.3)	
Definitely easier	27 (17.6)	11 (19.0)	75 (18.6)	

* Kruskal–Wallis ANOVA.

**Table 4:** Respondents’ perception of remote contact in PKU therapy.

Characteristics	1. Patients *n* (%N)	2. Caregivers of Patients over 18 Years Old *n* (%N)	3. Caregivers of Patients under 18 Years Old *n* (%N)	*p* * (Post Hoc Tests for Groups) **
**Number of Patients**	N = 153	N = 58	N = 403	
**Were you satisfied with the remote contact?**				**<0.001** (1 vs. 3, *p* < **0.001**)
Definitely	35 (22.9)	18 (31.1)	159 (39.5)	
Yes	41 (26.8)	17 (29.3)	120 (29.8)	
I do not know	66 (43.1)	21 (36.2)	111 (27.5)	
No	6 (3.9)	1 (1.7)	11 (2.7)	
Definitely not	5 (3.3)	1 (1.7)	2 (0.5)	
**Would you be interested in future remote contact?**				0.52
Definitely	36 (23.5)	9 (15.5)	99 (24.5)	
Yes	47 (30.7)	19 (32.8)	116 (28.8)	
I do not know	43 (28.1)	16 (27.6)	114 (28.3)	
No	17 (11.1)	10 (17.2)	62 (15.4)	
Definitely not	10 (6.6)	4 (6.9)	12 (3.0)	
**For PKU patients, can a remote visit replace direct contact with a specialist?**				0.10
Definitely	15 (9.8)	6 (10.4)	32 (7.9)	
Yes	51 (33.4)	17 (29.3)	129 (32.0)	
I do not know	23 (15.0)	5 (8.6)	42 (10.4)	
No	43 (28.1)	17 (29.3)	147 (36.5)	
Definitely not	21 (13.7)	13 (22.4)	53 (13.2)	
**In the case of other diseases, can you imagine remote contact with the doctor?**				0.27
Definitely	12 (7.8)	4 (6.9)	25 (6.2)	
Yes	33 (21.6)	13 (22.4)	76 (18.9)	
I do not know	28 (18.3)	7 (12.1)	51 (12.6)	
No	57 (37.3)	23 (39.6)	185 (45.9)	
Definitely not	23 (15.0)	11 (19.0)	66 (16.4)	

Statistically significant results are marked with boldface. * Kruskal–Wallis ANOVA. ** Post hoc test shown exclusively for significant differences.

**Table 5:** Self-reported satisfaction from remote contact with doctor/dietitian depending on contacts with doctor/dietitian and self-reported blood Phe levels before the pandemic lockdown.

	Were You Satisfied with the Remote Contact?	
	Patients Reporting Regular Contacts With dietitian/doctor before Lockdown vs. Others	Caregivers Reporting Regular Contacts With dietitian/doctor before Lockdown vs. Others
OR	7.92	5.25
95% CI	2.59–24.25	2.84–9.72
*p*	**<0.001**	**<0.001**
	Patients reporting recommended blood Phe levels before the pandemic lockdown vs. others	Caregivers reporting recommended blood Phe levels before the pandemic lockdown vs. others
OR	2.44	0.95
95% CI	1.26–4.70	0.61–1.47
*p*	**0.004**	0.40

Statistically significant results are marked with boldface.

**Table 6:** The impact of the time of travelling to a PKU-treating centre before the pandemic on believing that remote visits are a replacement for direct contact with a PKU specialist.

	How Long Does It Take to Get to Your Doctor?	
	Patients Travelling > 3 h to the Doctor vs. Other Patients	Caregivers Travelling > 3 h to the Doctor vs. Other Caregivers
OR	1.36	2.82
95% CI	0.48–3.48	1.42–5.61
*p*	0.58	**0.001**

Statistically significant results are marked with boldface.

**Table 7:** The relationship between reporting worse access to low-phenylalanine (low-Phe) products during the pandemic lockdown and self-reported changes in Phe levels.

	Was It Difficult to Stick to the Diet during the Pandemic Lockdown?	
	Patients Reporting Worse access to Low-Phe Products vs. Other Patients	Caregivers Reporting Worse access to Low-Phe Products vs. Other Caregivers
OR	0.94	1.61
95% CI	0.43–2.07	1.03–2.52
*p*	0.44	**0.02**

Statistically significant results are marked with boldface.

## Data Availability

The data presented in this study are available on reasonable request from the corresponding author.

## Abbreviations

PKU: phenylketonuria, Phe: phenylalanine, OR: odds ratio, IMD: inherited metabolic diseases.
